# The pervasive association between political ideology and COVID-19 vaccine uptake in Brazil: an ecologic study

**DOI:** 10.1186/s12889-023-16409-w

**Published:** 2023-08-23

**Authors:** Gabriel J. Seara-Morais, Thiago J. Avelino-Silva, Marcia Couto, Vivian I. Avelino-Silva

**Affiliations:** 1 Faculdade Israelita de Ciências da Saúde Albert Einstein, Hospital Israelita Albert Einstein, São Paulo, SP Brazil; 2 Laboratorio de Investigacao Medica Em Envelhecimento (LIM-66), Servico de Geriatria, Hospital das Clinicas HCFMUSP, Faculdade de Medicina, Universidade de Sao Paulo, São Paulo, SP Brazil; 3 Atlantic Fellowship for Equity in Brain Health at the Global Brain Health Institute, University of California, San Francisco, CA USA; 4 Department of Preventive Medicine, Faculdade de Medicina, Universidade de Sao Paulo, São Paulo, SP Brazil; 5 Department of Infectious and Parasitic Diseases, Faculdade de Medicina, Universidade de Sao Paulo, São Paulo, SP Brazil

**Keywords:** COVID-19, COVID-19 vaccines, Vaccination hesitancy, Politics, Socioeconomic factors, Mass vaccination, Human development, Political factors, Health policy, Public health

## Abstract

**Background:**

Despite the unequivocal benefits of vaccination, vaccine coverage has been falling in several countries in the past few years. Studies suggest that vaccine hesitancy is an increasingly significant phenomenon affecting adherence to vaccines. More recently, during the COVID-19 pandemic, political views have emerged as an additional influencing factor for vaccine hesitancy.

**Methods:**

In this ecologic study, we used information from publicly available databases to investigate the association between political ideology, depicted by the percentage of votes for the right-wing candidate Jair Bolsonaro in the presidential elections of 2018 and 2022, and COVID-19 vaccination in Brazilian municipalities. The primary endpoint was the COVID-19 vaccination index, calculated as the number of COVID-19 vaccine doses administered up to September 2022 divided by the number of inhabitants in each municipality. The analysis was conducted using Pearson correlation coefficients and linear regression models adjusted for HDI, the percentage of male voters, the percentage of voters who were older than 50 years old, and the percentage of voters with a middle school education or less. In addition, we explored whether the effect of the percentage of Bolsonaro voters on the COVID-19 vaccination index was modified in different quartiles of HDI using an interaction term.

**Results:**

Five thousand five hundred sixty-three Brazilian municipalities were included in the analysis. For both the 2018 and 2022 elections, the percentage of votes for Jair Bolsonaro was significantly and inversely associated with COVID-19 vaccine uptake after adjustment for the sociodemographic characteristics of the voters (change in mean vaccination index in 2018 for each 1% increase in Bolsonaro voters -0.11, 95% confidence interval [CI] -0.13 to -0.08, *p* < 0.001; change in mean vaccination index in 2022 for each 1% increase in Bolsonaro voters -0.09, 95% CI -0.11 to -0.07, *p* < 0.001). We also found a statistically significant interaction between the primary predictor of interest and HDI scores, with a more significantly detrimental effect of the right-wing political stance in municipalities in the lower HDI quartiles (interaction *p* < 0.001 for the first HDI quartile; *p* = 0.001 for the second HDI quartile).

**Conclusion:**

Our findings suggest that political ideologies have influenced COVID-19 vaccine hesitancy in Brazilian municipalities, affecting communities inequitably. The politicization of vaccines is a new challenge for vaccine programs. Strategies to face these challenges should include joint efforts from governments and civil society for a common public health goal.

## Introduction

Mass vaccination has had a crucial impact on worldwide public health in the past decades. In Brazil, the establishment of the National Immunization Program (Programa Nacional de Imunizações, PNI) in 1975 grounded the implementation of vaccines as an official and state-supported health policy [1]. As a result of coordinated efforts to expand access to vaccines, the mean vaccination coverage among Brazilian children younger than one year increased from 50% before the program to more than 90% by the late 1990s. Concurrently, a sharp reduction in cases and deaths due to vaccine-preventable diseases was registered in the country [1–3].

Despite the unequivocal benefits of vaccination, vaccine coverage has been falling in Brazil in the past few years, particularly since 2015 [4–6]. Non-adherence to vaccination recommendations can result from access barriers, including issues related to patient mobility and transportation, costs, working hours in vaccination clinics, shortage of supplies, and unawareness about recommended vaccines in distinct situations. However, in some cases, vaccines are voluntarily avoided or postponed after deliberate assessment and decision by the patient (or by a parent or legal guardian). This scenario has been referred to as “vaccine hesitancy” and has been observed in several developed countries in Europe, the USA, Canada, Japan, and Australia [7–9]. Some studies suggest that vaccine hesitancy is an increasingly significant phenomenon in Brazil, especially in subgroups with higher income and education [4, 6, 10–13].

Many studies have addressed COVID-19 vaccine hesitancy and acceptance in different countries. In a review published by Hassan et al. in 2021, COVID-19 vaccination acceptance was lower in Russia (55%), Italy (58%), the USA (67–69%), and Turkey (69%), while other countries such as the UK (86%) and China (91%) had higher acceptance rates [14]. In a systematic review including studies conducted in sub-Saharan African countries, the pooled COVID-19 vaccine acceptance was only 55% [15]. A more recent global review showed an overall acceptance of 65% [16]. Sociodemographic factors including younger age, female gender, lower socioeconomic status, Asian and black race/ethnicity, and Muslim or Buddhist religion were associated with lower acceptance of COVID-19 vaccines [17].

Interestingly, during the COVID-19 pandemic, political views have emerged as an additional influencing factor for vaccine hesitancy. For example, a study in the USA showed that counties with a higher percentage of votes for the Republican party had lower vaccination coverage and higher rates of COVID-19 cases and deaths [18]. In Brazil, former President Jair Bolsonaro refused to receive the COVID-19 vaccine and declared he would not give the vaccine to his daughter; moreover, he denied scientific evidence available at the time and made several declarations opposing recommendations from official health organizations during the pandemic [19–21]. These attitudes may have influenced overall adherence to the COVID-19 vaccination campaign in Brazil, particularly among Bolsonaro’s political supporters. In agreement with this hypothesis, two recent studies showed that Brazilian municipalities supporting Jair Bolsonaro in the 2018 elections were less compliant with social distancing measures in the first pandemic wave and had higher COVID-19 mortality rates, particularly during the second wave of the disease in 2021 [22, 23]. It is also plausible to assume that the detrimental influence of political ideology on vaccine acceptance and uptake might be heterogenous across different municipalities based on sociodemographic characteristics.

In this study, we used information from publicly available databases to investigate the association between political alignment, depicted by the percentage of Bolsonaro voters in the presidential elections of 2018 and 2022, and COVID-19 vaccination in Brazilian municipalities, adjusted for human development index (HDI) and basic demographic characteristics of voters.

## Methods

In this cross-sectional, ecologic study, the primary predictor of interest was the percentage of votes for Jair Bolsonaro in the first round of the 2018 and 2022 elections; the primary endpoint was the COVID-19 vaccination index, calculated as the number of COVID-19 vaccine doses administered up to September 2022 divided by the number of inhabitants in each municipality according to estimates from July 2021 [21–24]. We also obtained municipal-level data on the HDI, categorized into quartiles, with the highest quartile corresponding to municipalities with higher socioeconomic development, the percentage of male voters, the percentage of voters who were older than 50 years, and the percentage of voters with middle school education or less, using publicly available, de-identified databases [24, 25].

Characteristics of Brazilian municipalities were described using counts, percentages, medians, and interquartile ranges (IQR), overall and according to COVID-19 vaccination index quartiles. The association between the percentage of Bolsonaro voters and the vaccination index was investigated using linear regression models with robust standard errors, adjusted for HDI, the percentage of male voters, the percentage of voters who were older than 50 years old, and the percentage of voters with middle school education or less. In addition, we explored whether the effect of the percentage of Bolsonaro voters on the COVID-19 vaccination index was modified in different quartiles of HDI using an interaction term. We used scatter plots and Pearson coefficients to investigate the correlation between the percentage of Bolsonaro voters and the vaccination index according to quartiles of HDI. Municipalities with a vaccination index > 6 were considered outliers and excluded from correlation analyses. We used Stata 15.1 (StataCorp. College Station, TX: StataCorp LP) for all analyses, with a 0.05 significance level.

Per Resolution 510/2016 of the Brazilian National Health Council, our local Ethics Committee exempted our study from obtaining informed consent since we used exclusively publicly available, de-identified information.

## Results

Out of 5,570 Brazilian municipalities, 5,563 were included in the analysis based on the availability of data on the total population and number of COVID-19 vaccine doses.

Table 1 presents the overall sociodemographic characteristics of the municipalities included in the analysis, according to the COVID-19 vaccination index quartiles. More than 60% of all municipalities were located in the Northeast and Southeast regions of the country. The percentage of male voters was close to 50%, and the percentage of voters with middle school education or less was close to 53%, overall, and in each of the vaccination index quartiles. The percentage of voters who were older than 50 years old increased with increasing vaccination index quartiles. HDI distribution varied across COVID-19 vaccination index quartiles, with more municipalities with higher HDI in the higher vaccination index quartiles. The overall percentage of Bolsonaro voters was 41% in 2018 and 2022, with a lower percentage of votes in municipalities in the lower quartile of the COVID-19 vaccination index.

**Table 1:** Municipal-level characteristics according to quartiles of COVID-19 vaccination index

	All municipalities *N* = 5563	Vaccination index Quartile 1 *N* = 1391	Vaccination index Quartile 2 *N* = 1391	Vaccination index Quartile 3 *N* = 1390	Vaccination index Quartile 4 *N* = 1391
Region (%)					
North	448 (8)	366 (26)	59 (4)	17 (1)	6 (< 1)
Northeast	1792 (32)	541 (39)	487 (35)	433 (31)	331 (24)
Central-west	467 (8)	177 (13)	116 (8)	80 (6)	94 (7)
Southeast	1666 (30)	182 (13)	403 (29)	499 (36)	582 (42)
South	1190 (21)	125 (9)	326 (23)	361 (26)	378 (27)
Median population size (IQR)	11741 (5453–25769)	17584 (8965–34653)	15327 (7493–31589)	11343 (5467–24237)	5447 (3228–11609)
Percentage of male voters (IQR)	49 (48–51)	50 (48–51)	49 (48–50)	49 (48–50)	50 (49–51)
Percentage of voters older than 50 years old (IQR)	38 (34–42)	33 (30–37)	38 (34–41)	39 (35–49)	42 (37–45)
Percentage of voters with ≤ middle school education (IQR)	53 (46–59)	55 (48–60)	53 (46–59)	52 (45–59)	52 (46–58)
Human development index quartile (%)					
First	1397 (25)	582 (42)	332 (24)	277 (20)	206 (15)
Second	1386 (25)	427 (31)	364 (26)	293 (21)	302 (22)
Third	1412 (25)	250 (18)	342 (25)	384 (28)	436 (31)
Fourth	1359 (25)	131 (9)	348 (25)	434 (31)	446 (32)
Percentage of Bolsonaro voters in 1^st^ round 2018 (IQR)	41 (21–55)	32 (19–50)	41 (21–56)	44 (21–56)	45 (25–55)
Percentage of Bolsonaro voters in 1^st^ round 2022 (IQR)	41 (24–53)	35 (23–50)	41 (24–54)	42 (23–54)	44 (26–54)

*IQR* Interquartile range

Effect estimates obtained in multivariable models addressing the association between the percentage of Bolsonaro voters in 2018 and 2022 and the COVID-19 vaccination index adjusted for covariates and including an interaction term with HDI are presented in Table 2. For both elections, higher percentages of Bolsonaro voters were significantly associated with a lower vaccination index; moreover, the harmful effect of each percent increase in Bolsonaro voters was greater in the lowest quartile of HDI compared to the highest quartile in 2018; similarly, the harmful effect of each percent increase in Bolsonaro voters was greater in the first and second quartiles of HDI compared to the highest quartile in 2022. In 2018, each 1% increase in Bolsonaro voters was associated with a mean 0.11-unit reduction in the vaccination index for municipalities in the fourth HDI quartile and a mean 0.22-unit reduction in vaccination index for municipalities in the first HDI quartile (interaction *p*-value < 0.001). In 2022, each 1% increase in Bolsonaro voters was associated with a mean 0.09-unit reduction in the vaccination index for municipalities in the fourth HDI quartile, a mean 0.21-unit reduction in the vaccination index for municipalities in the first HDI quartile (interaction *p*-value < 0.001), and a mean 0.14-unit reduction in vaccination index for municipalities in the second HDI quartile (interaction *p*-value = 0.001).

**Table 2:** Multivariable models for COVID-19 vaccination index, including interaction between percentage of Bolsonaro voters and HDI

	2018 election		2022 election	
Variables	Coefficient (95% CI)	*p*-value	Coefficient (95% CI)	*p*-value
Change in mean vaccination index for each 1% increase of male voters	0.39 (-0.44 to 1.21)	0.357	1.20 (0.37 to 2.02)	0.005
Change in mean vaccination index for each 1% increase of voters with ≤ middle school education	-0.59 (-0.82 to -0.35)	< 0.001	-0.49 (-0.73 to -0.26)	< 0.001
Change in mean vaccination index for each 1% increase of voters older than 50 years old	4.97 (4.66–5.28)	< 0.001	4.78 (4.48 to 5.09)	< 0.001
Change in mean vaccination index for each 1% increase of Bolsonaro voters^a^	-0.11 (-0.13 to -0.08)	< 0.001	-0.09 (-0.11 to -0.07)	< .001
Change in mean vaccination index for each category of HDI^b^				
First	-0.56 (-0.66 to -0.47)	< 0.001	-0.47 (-0.56 to -0.39)	< 0.001
Second	-0.28 (-0.34 to -0.21)	< 0.001	-0.24 (-0.31 to -0.18)	< 0.001
Third	-0.12 (-0.18 to -0.06)	< 0.001	-0.09 (-0.15 to -0.04)	0.001
Fourth	Reference	-	-	-
Interaction term: Mean difference in the effect of each 1% increase of Bolsonaro voters for each HDI category				
First	-0.11 (-0.14 to -0.07)	< 0.001	-0.12 (-0.15 to -0.08)	< 0.001
Second	-0.02 (-0.05 to 0.01)	0.216	-0.05 (-0.08 to -0.02)	0.001
Third	0.02 (-0.01 to 0.05)	0.214	0.00 (-0.03 to 0.03)	0.828
Fourth	Reference	-	Reference	-

*HDI* Human development index

^a^Among municipalities in the fourth (higher) human development index quartile

^b^Among municipalities with mean percentage of Bolsonaro voters

The percentage of male voters had no statistically significant effect on the vaccination index in the model, including the 2018 election results; however, higher percentages of male voters were associated with a higher COVID-19 vaccination index in the model, including the 2022 election results. Higher percentages of voters with middle school education or less were significantly associated with a lower vaccination index in both models, whereas higher percentages of voters older than 50 years old were significantly associated with a higher vaccination index in both models. Finally, lower quartiles of HDI were significantly associated with lower vaccination index in both models.

**Fig. 1:**
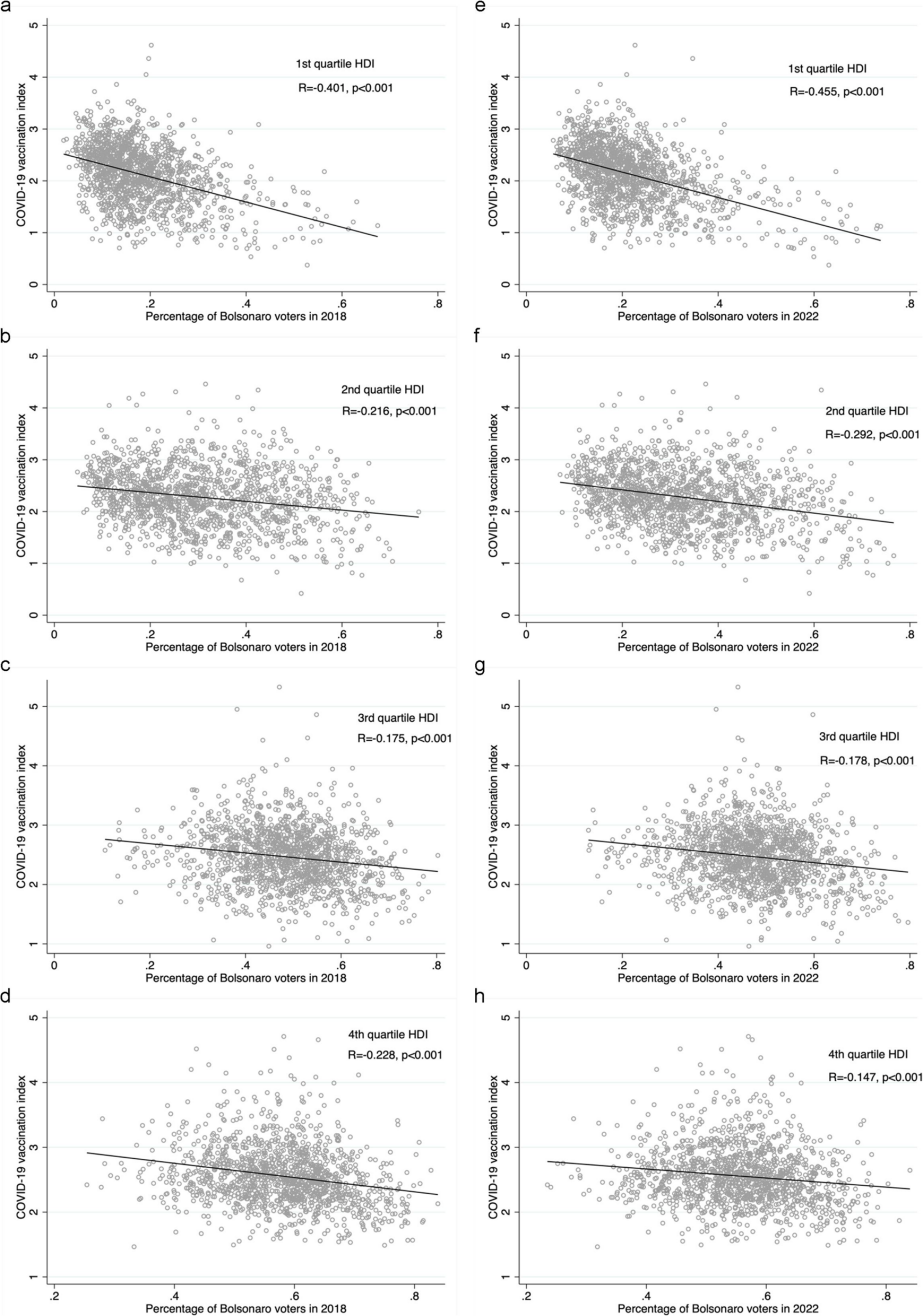
Correlations between percentage of Bolsonaro voters and COVID-19 vaccination in Brazilian municipalities, by HDI quartiles

The correlations between the percentage of Bolsonaro voters in 2018 and 2022 and the COVID-19 vaccination index in Brazilian municipalities, according to human development index quartiles, are presented in Fig. 1. As observed in the multivariable models, the inverse correlation was stronger in municipalities in the lowest HDI quartile for both the 2018 and 2022 elections.

## Discussion

In this cross-sectional ecologic study including Brazilian municipalities as units of observation, we showed that political ideology, depicted by the percentage of votes for the right-wing candidate Jair Bolsonaro, is significantly and inversely associated with COVID-19 vaccine uptake after adjusting for sociodemographic characteristics of the voters. We also showed a statistically significant interaction between the percentage of Bolsonaro voters and HDI scores, with a more significantly detrimental effect of the right-wing political stance in municipalities in the lower HDI quartile.

Our findings parallel results from an ecological study conducted in the USA, which demonstrated that counties with higher percentages of votes for the Republican party in the 2016 presidential election had lower COVID-19 vaccination coverage. Moreover, this study showed that vaccination was a mediator in the association between the percentage of votes for Donald Trump and COVID-19 cases and deaths [18]. Notably, Donald Trump and Jair Bolsonaro, both in line with a far-right wing denialist rhetoric, and respective heads of state of the United States and Brazil during the most challenging periods of the COVID-19 pandemic, expressed similar attitudes towards non-pharmacological prevention strategies and COVID-19 vaccines [26, 27]. For instance, both presidents opposed the adoption of facial masks and physical distancing and mobility restrictions [28–32]; both repelled the implementation of vaccines produced in China [33–35]; both manifested mistrust regarding COVID-19 vaccines [36, 37]; and both concealed information regarding their COVID-19 vaccination status [38, 39]. Our results are also supported by a recent survey study published by Paschoalloto et al., showing that willingness to be vaccinated for COVID-19 is strongly associated with political orientation [40]. Our study provides additional evidence on the pervasive influence political ideologies can have on COVID-19 vaccination in Brazil and shows that municipalities in more vulnerable socioeconomic conditions seem even more susceptible to this effect. It is further disquieting that lower vaccination rates will likely lead to a higher disease burden in these municipalities, exacerbating prevailing social disparities.

Significantly, while the impact of political ideologies on the consequences of the pandemic in Brazil is unequivocal, it must be emphasized that President Jair Bolsonaro's rhetoric was not the sole cause of vaccine hesitancy or other deleterious effects of COVID-19 in the nation. Persistent social and health disparities, exacerbated by a decrease in social policy funding over the past years, fostered an environment ripe for a calamitous situation within Brazilian public health. A sizable and influential faction of Bolsonaro's supporters, encompassing agriculture industry representatives, evangelicals, and prosperous entrepreneurs, supported his positions throughout the pandemic, contesting the recommendations of research scientists, international health organizations, and public health officials. Therefore, a complex political landscape contributed to a mitigated comprehensive response to the pandemic in Brazil, including immunization efforts [21, 22, 41].

While COVID-19 vaccine coverage is more directly relevant to the middle and long-term control of COVID-19, uptake of other vaccines has also been indirectly affected by the recent pandemic scenario [42]. Several studies suggest that adherence to routine vaccines has dropped since the onset of the COVID-19 pandemic [43–47]. Disruptions in healthcare services likely intensified barriers to vaccination in several settings. Nonetheless, it is also possible that skepticism towards COVID-19 vaccines built up distrust and hesitancy towards other vaccines, as suggested by a previous study on the influenza vaccine [48]. Consequently, escalating challenges related to reductions in vaccine coverage, including outbreaks of vaccine-preventable diseases, might happen even after the re-establishment of routine care in vaccine clinics affected by the pandemic. Reluctant attitudes toward the COVID-19 vaccine may have reinforced the growing phenomenon of vaccine hesitancy.

Our study had limitations. First, we used HDI data from 2010 since more recent municipal-level information was unavailable from official sources. Second, age and schooling were analyzed as binary variables since more granularity or individual-level data could not be obtained from available datasets. Third, we used an ecologic design, which could be prone to ecologic fallacy and confounding. Even so, we were able to include data from most Brazilian municipalities and investigate interactions between the percentage of Bolsonaro voters and HDI on the COVID-19 vaccination index, adjusted for other sociodemographic covariates. To our knowledge, this is the first study addressing such an association in Brazil. Furthermore, we used data from both the 2018 and 2022 elections and found similar results, supporting that political views before and after the COVID-19 vaccine rollout were associated with our vaccination index.

## Conclusion

There are several implications for our results. For over two decades, Brazil had been able to provide a robust public policy within the PNI, achieving high vaccination coverage for most vaccines and establishing a widespread culture of vaccination [1, 49]. However, in recent years the rates of vaccination have been dropping, with consequences that included a recent outbreak of measles in the state of Sao Paulo, in 2019 [50, 51]. While barriers to vaccine access should still be confronted, vaccine hesitancy seems to be an increasingly concerning issue, enhanced by political ideologies and potentially affecting communities inequitably [35, 52]. Therefore, our study highlights what may be the beginning of a new scenario with unforeseen challenges for the PNI: the politicization of vaccines. Until recently, the Program recorded fairly high adherence to vaccines recommended in the national immunization calendar, and subsequent widespread reductions in coverage appeared unrelated to political affiliations or beliefs. However, in the current political scenario in Brazil, vaccines have shifted from 'a health issue' to 'a political issue'. This could be the outcome of rhetorical disputes over the pandemic, which went beyond health and sanitation issues and strengthened the contemporary clash of worldviews on human relations, society's organization, the role of governments, and the economy [53, 54]. Therefore, initiatives to address these difficulties should include collaborative efforts by governments and civil society toward a common goal that prioritizes public health regardless of individual political preferences.

## Data Availability

This study used publicly available, de-identified data only. Data sources are provided in the reference list (references [21–24]).

## Abbreviation

**PNI**: National Immunization Program (Programa Nacional de Imunizações)
